# Real-World Clinical and Healthcare Resource Burden Among Burosumab-Naïve Patients With Familial Hypophosphatemia

**DOI:** 10.1210/jendso/bvae185

**Published:** 2024-10-24

**Authors:** Erik A Imel, Zhiyi Li, Heather M Heerssen, Nicole Princic, Hana Schwartz, Yang Zhao, Kathryn M Dahir

**Affiliations:** Departments of Medicine and Pediatrics, Indiana University School of Medicine, Indianapolis, IN 46202, USA; Kyowa Kirin, Inc., Princeton, NJ 08540, USA; Kyowa Kirin, Inc., Princeton, NJ 08540, USA; Merative, Ann Arbor, MI 48108, USA; Merative, Ann Arbor, MI 48108, USA; Kyowa Kirin, Inc., Princeton, NJ 08540, USA; Department of Endocrinology, Vanderbilt University Medical Center, Nashville, TN 37232, USA

**Keywords:** disease burden, familial hypophosphatemia, healthcare costs, healthcare resource utilization, X-linked hypophosphatemia (XLH), burosumab

## Abstract

**Objective:**

To examine the real-world clinical and healthcare resource burden of familial hypophosphatemia (FH).

**Methods:**

In a retrospective, observational cohort study using MarketScan claims data from 2017 to 2021, clinical characteristics and healthcare resource utilization (HCRU) and costs were compared between burosumab-naïve pediatric and adult patients with ≥ 1 FH diagnosis code and matched controls without FH. Patient characteristics were evaluated at baseline, and disease characteristics, HCRU, and costs were evaluated over a 12-month follow-up period. Outcomes were analyzed descriptively. Costs were additionally analyzed using multivariate regression models.

**Results:**

Overall, 570 patients with FH and 1710 non-FH matched controls were included. Approximately 10% of study participants were aged < 18 years. Patients with FH had 7.8-fold higher mean baseline comorbidity (Charlson Comorbidity Index). The prevalence of morbidities over the 12-month follow-up period was higher in patients with FH than controls, including renal disease (33% vs 3%), arthralgia (25% vs 10%), osteoarthritis (17% vs 6%), and delayed growth/walking difficulty (16% vs 2%; all *P* < .001). All-cause HCRU was significantly greater for patients with FH than controls over follow-up, including the proportion of patients with at least one inpatient admission (60% vs 4%), outpatient emergency room visit (52% vs 16%), and outpatient pharmacy prescription (96% vs 71%; all *P* < .001). The mean annual total healthcare cost per patient was also 22.6-fold higher for patients with FH than controls (adjusted cost difference = $129 643; *P* < .001). Differences were apparent across all age groups.

**Conclusion:**

Compared with non-FH matched controls, burosumab-naïve patients with FH experienced multiple morbidities and had substantially higher HCRU and costs.

Familial hypophosphatemia (FH) represents a group of rare genetic disorders that are typically due to renal phosphate wasting, resulting in chronic hypophosphatemia and progressive musculoskeletal complications [1-3]. X-linked hypophosphatemia (XLH) is the most common form of FH, accounting for approximately 80% of FH cases and affecting up to one in 20 000 individuals globally [1-4]. XLH arises through pathogenic variants in the *PHEX* gene that are inherited in an X-linked dominant pattern or present as *de novo* variants, causing increased fibroblast growth factor 23 (FGF23) gene expression; elevations in circulating FGF23 levels lead to impaired renal phosphate conservation [1, 3-7]. While other, rarer inheritance patterns for FH result from different gene variants and mechanisms, most diagnoses of FH in the real world are likely to represent XLH, given its preponderance [2, 6-9].

The lifelong hypophosphatemia associated with XLH, as well as other effects of *PHEX* variants, results in defective bone mineralization and tooth formation, leading to the accumulation of morbidities over a lifetime that can include rickets, limb deformities, and short stature in children; fractures, arthritis, enthesopathy, and spinal stenosis in adults; and osteomalacia, hearing loss, endodontic infections, and periodontitis at all ages [1, 3, 4, 6, 8, 10, 11]. Both children and adults with the disorder experience a substantial and persistent disease burden, including pain, impaired physical functioning, fatigue, muscle weakness, and the need for orthopedic surgery [12-14]. XLH has a broad detrimental impact on patients’ quality of life, affecting physical, dental, and psychosocial well-being [3, 6, 12-16].

Disease management often includes care by numerous medical professionals, including pediatricians, endocrinologists, rheumatologists, nephrologists, pain management specialists, audiologists, neurologists, rehabilitation specialists, orthopedic surgeons, and dentists [3, 10, 17, 18]. As the symptoms and morbidities associated with XLH are broad and progressive with age, individualized management plans are necessary [1, 3, 17].

Since the late 1970s, pharmacologic doses of oral phosphate salts and active vitamin D (1,25(OH)_2_D) have been established as conventional treatment for XLH [1-3, 6, 7]. These therapies target the phosphate and active vitamin D deficiencies consequent to FGF23 excess, rather than addressing the disease-causing elevation in FGF23 itself [1, 3]. Potential adverse effects of conventional treatment include nephrocalcinosis, hypercalcemia, hypercalciuria, hyperparathyroidism, and gastrointestinal issues, as well as exacerbated hypophosphatemia through elevation of circulating FGF23 levels and parathyroid hormone [2, 3, 5, 10]. In adults with XLH, conventional treatment is typically recommended only in patients who are symptomatic because of its associated adverse effects and high burden of administration [1, 3, 6, 10, 19]. Access can also be an issue, as these therapies do not have regulatory approval for XLH in the United States and thus may not be covered under health insurance plans due to a misconception of being “supplements” [1, 4].

In 2018, burosumab, an antibody that inhibits FGF23 activity directly, became the first and only US Food and Drug Administration–approved treatment to date for XLH, with approval extended to patients 6 months of age and older in 2019 [6, 7, 20, 21]. Clinical trials have demonstrated the efficacy of burosumab in pediatric and adult patients in terms of several outcomes, including serum phosphate control, improvements in bowing of legs, fracture healing, and improved physical function [3, 22-28]. Given its demonstrated efficacy, burosumab has been incorporated into multiple clinical practice recommendations for XLH [3, 6, 10, 18, 19, 29]. However, the current use of burosumab in clinical practice, particularly in adults, remains relatively low, and access can be limited [3, 5, 30].

Although the clinical impact of XLH and frequent need for clinical intervention are well recognized [3, 6, 12, 31-33], few studies to date have attempted to determine the healthcare resource burden associated with XLH or other forms of FH. A recent literature review identified the substantial and lifelong burden associated with XLH, including healthcare resource utilization (HCRU) across categories of pharmacological therapy, management of pain and mobility, orthopedic procedures, morbidities associated with XLH or conventional treatment, and productivity [5]. The analysis showed that up to 60% of adults with XLH sustained fractures and up to 60% underwent corrective orthopedic procedures [5]. To further expand our understanding of the clinical and healthcare resource burden of FH in the United States, this real-world study was conducted to evaluate the patient and treatment characteristics, HCRU, and healthcare costs of burosumab-naïve patients with FH compared with a non-FH matched control population.

## Materials and Methods

### Study Design and Data Source

This was a US retrospective claims database study. Administrative claims data were obtained from the Merative MarketScan Commercial, Medicare, and Dental databases between January 1, 2017, and December 31, 2021 (data from the Dental database were only available to December 31, 2020). These databases contain a longitudinal view of inpatient and outpatient services, prescription and office/outpatient-administered drugs, and costs, as well as detailed enrollment information; data are de-identified and compliant with the Health Insurance Portability and Accountability Act (HIPAA) to protect patient privacy [34]. The databases are nationally distributed and are representative of the approximate 55% of the US population with employer-sponsored health insurance.

**Figure 1.**
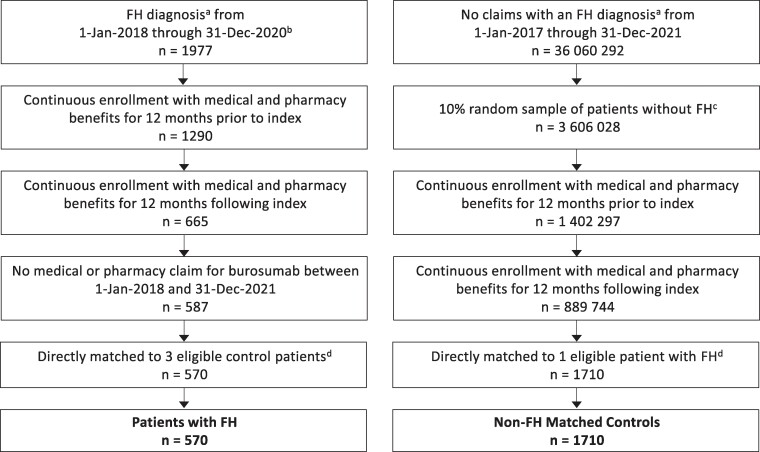
Patient selection for study cohorts. ^a^Inpatient or outpatient claim with an FH diagnosis. ^b^Index date of patients with FH corresponded to the earliest date of FH diagnosis occurrence in the system. ^c^Index date of controls was randomly assigned based on the index dates of patients with FH. ^d^Direct matching with a 1:3 ratio of patients with FH to non-FH controls was based on age, sex, geographic region, payer, and index year. Abbreviation: FH, familial hypophosphatemia.

Patients included in the FH cohort had at least one diagnosis of FH based on the presence of an International Classification of Diseases, Tenth Revision, Clinical Modification (ICD-10-CM) diagnosis code for FH (E83.31; synonyms: familial hypophosphatemia, vitamin D-resistant osteomalacia, vitamin D-resistant rickets) during the index identification period from January 1, 2018, through December 31, 2020 (Fig. 1). Patients treated with burosumab between January 1, 2018, and December 31, 2021, were excluded. The index date for patients in the FH cohort was the earliest date of FH diagnosis during the index identification period. For the non-FH matched cohort, an initial pool of controls was selected using a random sample (10%) in the MarketScan Commercial or Medicare databases who had no claims involving an ICD-10-CM diagnosis code for FH during the entire study period (Fig. 1). The index date was randomly assigned for all potential controls based on the index dates of patients with FH to maintain a consistent distribution. Patients in both cohorts were required to have a minimum of 12 months of continuous enrollment in a health insurance plan with medical and pharmacy benefits both pre- and post-index, allowing for a 12-month baseline period and a 12-month follow-up period. The cohorts were directly matched with a 1:3 ratio based on age, sex, geographic region, payer, and index year; data for race were not available. For dental outcomes, a 1:2 matching ratio was used because smaller numbers of patients were able to be linked to the MarketScan Dental database.

All study outcomes were compared between patients with FH and non-FH matched controls. Subgroup analyses were also conducted for cohorts stratified by age group on the index date.

### Outcomes and Measures

Baseline demographic and clinical characteristics were compared between patients with FH and non-FH matched controls. Demographic characteristics were measured on the index date. Charlson Comorbidity Index (CCI) [35, 36], a measure of baseline comorbidity, was determined during the 12-month pre-index period.

Treatment characteristics, morbidities and other complications, and all-cause HCRU and costs were examined over the 12-month follow-up period; dental HCRU and costs were also determined for the subset of patients and controls linked to the MarketScan Dental database. Medications were identified via national drug codes and Healthcare Common Procedure Coding System (HCPCS) codes. Morbidities and other complications were identified by the presence of at least one ICD-10-CM diagnosis code for the given condition. All-cause HCRU and costs were reported by type of service over the follow-up period; healthcare costs were also determined for the pre-index period to provide a baseline value prior to the index date. Healthcare costs and dental costs were based on reimbursed amounts of adjudicated claims, including health plan payments and patients’ out-of-pocket payments. The cost for healthcare services provided under capitated arrangements was estimated using payment proxies, which were computed based on paid claims at the procedure level using the MarketScan Commercial and Medicare databases; dental claims did not have payment proxies, but capitation in dental claims is uncommon (< 1%) in the MarketScan Dental database. All costs were inflated to 2021 US dollars using the Medical Care Component of the consumer price index.

### Statistical Analyses

Outcomes were analyzed descriptively and compared between patients with FH and non-FH matched controls. Continuous variables were summarized with means, SD, and medians, and categorical variables were described with counts and percentages. T-tests and chi-square tests were used to evaluate the statistical significance (*P* < .05) of differences for continuous and categorical variables, respectively.

For the cost analyses, mean costs were calculated for 4 annual all-cause cost outcomes, adjusting for age, sex, and insurance plan type: total healthcare costs, inpatient admission costs, outpatient service costs, and outpatient pharmacy prescription costs. Multivariate analysis was conducted to compare all-cause healthcare costs between patients with FH and non-FH controls. A two-part model was used: part 1 was a logistic regression analysis that modeled the probability of a nonzero cost, and part 2 was a generalized linear model with a gamma distribution and log link that estimated the adjusted cost values. A multivariate logistic regression model was also used to compare the odds of opioid use among patients with FH vs non-FH controls.

## Results

Among 1977 patients with a diagnosis of FH between January 2018 and December 2020, 587 patients were eligible for study participation and were directly matched 1:3 to eligible non-FH controls, leading to a total of 570 patients with FH and 1710 non-FH matched controls being included in the study (Fig. 1). Notably, burosumab use resulted in exclusion of 78 patients, comprising 40 adults aged ≥ 18 years and 38 children aged < 18 years. Approximately 20% of patients with FH (115 out of 570) and 18% of controls (313 out of 1710) could be linked to the MarketScan Dental database, and a 1:2 matching ratio led to a study population of 71 patients with FH and 142 controls for the dental analyses. For the subgroup analysis of study participants stratified by age group, the proportion of patients in each age category is shown in Table 1.

**Table 1.** Baseline demographic and clinical characteristics for patients with FH and non-FH matched controls

Characteristic	Patients with FH n = 570	Non-FH matched controls n = 1710	*P* value
**Age, years, mean (SD)**	47.2 (19.9)	46.2 (18.3)	.249
**Age category, years, n (%)**			1.000
0-11	30 (5.3%)	90 (5.3%)	
12-17	29 (5.1%)	87 (5.1%)	
18-29	49 (8.6%)	147 (8.6%)	
30-39	70 (12.3%)	210 (12.3%)	
40-49	95 (16.7%)	285 (16.7%)	
50-64	220 (38.6%)	660 (38.6%)	
65+	77 (13.5%)	231 (13.5%)	
**Female sex, n (%)**	325 (57.0%)	975 (57.0%)	1.000
**Geographic region, n (%)*^a^***			1.000
Northeast	77 (13.5%)	231 (13.5%)	
North Central	171 (30.0%)	513 (30.0%)	
South	254 (44.6%)	762 (44.6%)	
West	66 (11.6%)	198 (11.6%)	
Unknown	2 (0.4%)	6 (0.4%)	
**Payer, n (%)**			1.000
Commercial	493 (86.5%)	1479 (86.5%)	
Medicare Supplemental	60 (10.5%)	180 (10.5%)	
Medicare Advantage	17 (3.0%)	51 (3.0%)	
**Physician specialty closest to index date, n (%)*^b^***			—
Acute care hospital	184 (32.3%)	N/A	
Internal medicine	50 (8.8%)	N/A	
Family practice	39 (6.8%)	N/A	
Emergency medicine	27 (4.7%)	N/A	
Radiology	28 (4.9%)	N/A	
Other/Unknown	242 (42.5%)	N/A	
**Index year, n (%)**			1.000
2018	169 (29.7%)	507 (29.7%)	
2019	133 (23.3%)	399 (23.3%)	
2020	150 (26.3%)	450 (26.3%)	
2021	118 (20.7%)	354 (20.7%)	
**Charlson Comorbidity Index*^c^***			
Mean (SD)	1.87 (2.58)	0.24 (0.88)	<.001
Median (IQR)	1 (3)	0 (0)	
Charlson Comorbidity Index ≥ 2, n (%)	212 (37.2%)	83 (4.9%)	<.001
**Baseline all-cause total healthcare costs, mean (SD)*^d^***	$68 761.25 ($159 267.12)	$3699.27 ($14 636.67)	<.001

Abbreviations: FH, familial hypophosphatemia; IQR, interquartile range; N/A, not available.

^*a*^The overall geographic breakdown of the MarketScan population in 2020 was 20.2% from the Northeast, 19.8% from the North Central, 46.3% from the South, 13.3% from the West, and 0.4% Unknown.

^*b*^Physician specialty was determined by the provider code on the physician office visit within 30 days of index.

^*c*^Components of the Charlson Comorbidity Index score included myocardial infarction, congestive heart failure, peripheral vascular disease, cerebrovascular disease, dementia, chronic pulmonary disease, rheumatologic disease, peptic ulcer disease, mild liver disease, diabetes (mild to moderate), diabetes with chronic complications, hemiplegia or paraplegia, renal disease, moderate or severe liver disease, human immunodeficiency virus, metastatic solid tumor, and any other malignancy.

^*d*^All costs were inflated to 2021 US dollars using the Medical Care Component of the consumer price index.

### Patient Characteristics

Per the study design, the demographic characteristics of the 2 study cohorts were similar (Table 1). Overall, 10.4% of study participants were aged < 18 years and 57.0% were female.

Patients with FH had a greater comorbidity burden, as indicated by a 7.8-fold higher mean baseline CCI than controls (mean CCI 1.87 vs 0.24; *P* < .001), with a higher proportion of diagnoses for every CCI component condition (data not shown). Mean CCI increased with age in adulthood (eg, 18-29 years: 0.63 for patients with FH vs 0.07 for controls; 30-39 years: 0.89 vs 0.10; 65+ years: 3.99 vs 0.58; all *P* < .001) (Supplementary Table S1) [37].

Mean baseline all-cause total healthcare costs during the 12-month pre-index period were 18.6-fold higher for patients with FH compared with controls ($68 761.25 vs $3699.27; *P* < .001).

### Treatment Characteristics

As expected, treatment with vitamin D (including both active vitamin D and other prescribed forms of vitamin D) and/or phosphate salts was more frequent over the follow-up period among patients with FH compared with controls (vitamin D prescriptions: 16.7% vs 1.5%; phosphate salt prescriptions: 7.9% vs 0.0%; both *P* < .001) (Table 2). Conventional treatment with active vitamin D (calcitriol, paricalcitol, doxercalciferol, or dihydrotachysterol) and/or phosphate salts was prescribed in 14.6% (83 out of 570) of patients with FH, while nonactivated forms of vitamin D (ergocalciferol or cholecalciferol) were prescribed in 6.5% (37 out of 570) of these patients. Vitamin D in any form was most commonly prescribed among juvenile patients with FH (aged 0-11 or 12-17 years), while phosphate salts were not prescribed in any participant aged ≥ 65 years (Supplementary Table S2) [37].

**Table 2.** Treatment characteristics during follow-up for patients with FH and non-FH matched controls

	Patients with FH n = 570	Non-FH matched controls n = 1710	*P* value
**Conventional treatment (active vitamin D and/or phosphate salts) and/or nonactivated (native) vitamin D, n (%)**	116 (20.4%)*^a^*	26 (1.5%)	N/A
**Vitamin D (any form), n (%)*^b^***			
Patients with a vitamin D prescription, n (%)	95 (16.7%)*^c^*	26 (1.5%)	**<**.**001**
Vitamin D prescriptions among patients with a prescription, mean (SD)	4.0 (3.2)	3.6 (3.5)	.556
**Phosphate salts, n (%)*^d^***			
Patients with an oral phosphate prescription, n (%)	45 (7.9%)	0 (0.0%)	**<**.**001**
Oral phosphate prescriptions among patients with a prescription, mean (SD)	3.6 (3.1)	N/A	N/A
**Antidepressants**			
Patients with an antidepressant prescription, n (%)	232 (40.7%)	208 (12.2%)	**<**.**001**
Antidepressant prescriptions among patients with a prescription, mean (SD)	8.2 (6.8)	5.9 (4.7)	**<**.**001**
**Nonopioid pain medications*^e^***			
Patients with a pain medication prescription, n (%)	258 (45.3%)	273 (16.0%)	**<**.**001**
Pain medication prescriptions among patients with a prescription, mean (SD)	4.9 (5.1)	2.8 (3.0)	**<**.**001**
**Opioid pain medications**			
Patients with an opioid prescription, n (%)	258 (45.3%)	200 (11.7%)	**<**.**001**
Opioid prescriptions among patients with a prescription, mean (SD)	4.2 (5.4)	2.3 (3.1)	**<**.**001**

Mean number of prescriptions per category was determined by the number of prescriptions for each patient with the given prescription type and then averaged.

Abbreviations: FH, familial hypophosphatemia; N/A, not available.

^*a*^In the FH cohort, 83 (14.6%) of patients had prescriptions for conventional treatment (active vitamin D and/or phosphate salts). The use of conventional treatment was not applicable for non-FH matched controls.

^*b*^Vitamin D included active vitamin D (calcitriol, paricalcitol, doxercalciferol, and dihydrotachysterol) and other prescribed forms of vitamin D (ergocalciferol and cholecalciferol).

^*c*^Among patients with FH with prescriptions for vitamin D, 58 (10.2%) had a prescription for active vitamin D and 37 (6.5%) had a prescription for nonactivated (native) vitamin D.

^*d*^Phosphate salts included potassium phosphate; potassium phosphate and sodium phosphate; potassium phosphate, monobasic and sodium phosphate, monobasic, anhydrous; sodium biphosphate and sodium phosphate; and sodium phosphate dibasic and sodium phosphate monobasic.

^*e*^Prescribed nonopioid pain medications evaluated in the study were compiled based on expected treatments for pain management in the patient population, including acetaminophen, aspirin, nonsteroidal anti-inflammatory drugs, anticonvulsants, muscle relaxants, and local anesthetics (eg, lidocaine) [38, 39].

Patients with FH also had greater medication utilization across other categories of interest compared with controls, including antidepressants (40.7% vs 12.2%; *P* < .001), nonopioid pain medications (45.3% vs 16.0%; *P* < .001), and opioid pain medications (45.3% vs 11.7%; *P* < .001) (Table 2). In the multivariate analysis, patients with FH were more likely to have a prescription for an opioid pain medication than controls (adjusted odds ratio = 6.3; *P* < .001) (Supplementary Table S3) [37].

### Morbidities and Other Complications

Morbidities and other complications were more common in patients with FH compared with controls, with prevalence differing significantly (Table 3). The most common morbidities in patients with FH were renal disease (including impaired renal function, renal insufficiency, chronic kidney disease, and chronic nephritic syndrome but excluding nephrocalcinosis; 33.2% vs 3.0% among controls), arthralgia (25.4% vs 10.3%), osteoarthritis (17.4% vs 6.5%), delayed growth/difficulty in walking (16.5% vs 1.6%), muscle weakness (10.7% vs 1.1%), and fracture (9.8% vs 1.6%; all *P* < .001 vs controls) (Table 3). The prevalence of morbidity diagnosis codes generally increased with age (Supplementary Table S4) [37]. The presence of lower limb deformity (genu varum or genu valgum) was more commonly coded among juvenile patients with FH than controls, but did not differ significantly between adult patients and controls. In contrast, fracture prevalence differed between adult patients and controls, but not between pediatric patients and controls.

**Table 3.** Morbidities and general comorbidities during follow-up for patients with FH and non-FH matched controls

	Patients with FH n = 570	Non-FH matched controls n = 1710	*P* value
**Morbidities, n (%)**			
Impaired renal function/renal insufficiency/chronic kidney disease, chronic nephritic syndrome	189 (33.2%)	52 (3.0%)	<.001
Arthralgia	145 (25.4%)	176 (10.3%)	<.001
Osteoarthritis	99 (17.4%)	112 (6.5%)	<.001
Delayed growth/difficulty in walking	94 (16.5%)	28 (1.6%)	<.001
Muscle weakness	61 (10.7%)	19 (1.1%)	<.001
Fracture	56 (9.8%)	27 (1.6%)	<.001
Hearing loss	34 (6.0%)	19 (1.1%)	<.001
Myalgia	26 (4.6%)	23 (1.3%)	<.001
Enthesopathy	24 (4.2%)	30 (1.8%)	.001
Spinal stenosis	24 (4.2%)	6 (0.4%)	<.001
Hyperparathyroidism	21 (3.7%)	2 (0.1%)	<.001
Rickets	20 (3.5%)	0 (0.0%)	<.001
Genu varum or genu valgum (lower limb deformity)	20 (3.5%)	0 (0.0%)	<.001
Kidney stone	19 (3.3%)	10 (0.6%)	<.001
Dental defects	10 (1.8%)	9 (0.5%)	.013
Short stature	6 (1.1%)	1 (0.1%)	.001
Nephrocalcinosis	4 (0.7%)	0 (0.0%)	.004
Osteomalacia	4 (0.7%)	0 (0.0%)	.004
**General comorbidities, n (%)**			
Hypertension	316 (55.4%)	297 (17.4%)	<.001
Obesity	194 (34.0%)	281 (16.4%)	<.001
Depression	128 (22.5%)	95 (5.6%)	<.001

Abbreviation: FH, familial hypophosphatemia.

General comorbidities were also significantly more common among patients with FH than controls (hypertension: 55.4% vs 17.4%; obesity: 34.0% vs 16.4%; depression: 22.5% vs 5.6%; all *P* < .001) (Table 3) and increased in prevalence with increasing age (data not shown).

### All-Cause HCRU and Costs

All-cause HCRU during the 12-month follow-up period was significantly greater for patients with FH compared with controls across all health service categories, including inpatient admissions (60.4% vs 4.3%; *P* < .001), outpatient emergency room visits (51.6% vs 15.7%; *P* < .001), outpatient office visits (97.5% vs 77.9%; *P* < .001), other outpatient visits (eg, laboratory tests, imaging; 99.8% vs 84.8%; *P* < .001), and outpatient pharmacy prescriptions (95.8% vs 71.1%; *P* < .001) (Table 4).

**Table 4.** All-cause HCRU during follow-up for patients with FH and non-FH matched controls

	Patients with FH n = 570	Non-FH matched controls n = 1710	*P* value
**Inpatient**			
Patients with an admission, n (%)	344 (60.4%)	74 (4.3%)	<.001
Inpatient admissions among all patients, mean (SD)	1.2 (1.8)	0.1 (0.3)	<.001
Median	1.0	0.0	
Inpatient admissions among patients with an admission, mean (SD)	2.0 (2.0)	1.3 (0.7)	.002
Median	1.0	1.0	
Length of stay (in days) per admission, mean (SD)	7.5 (9.3)	4.6 (4.2)	.009
Median	5.0	3.0	
**Outpatient**			
ER visits			
Patients with an ER visit, n (%)	294 (51.6%)	269 (15.7%)	<.001
ER visits among all patients, mean (SD)	1.3 (2.1)	0.2 (0.6)	<.001
Median	1.0	0.0	
ER visits among patients with a visit, mean (SD)	2.5 (2.4)	1.4 (0.8)	<.001
Median	2.0	1.0	
Outpatient office visits			
Patients with an office visit, n (%)	556 (97.5%)	1332 (77.9%)	<.001
Office visits among all patients, mean (SD)	11.6 (9.1)	2.9 (3.7)	<.001
Median	9.5	2.0	
Office visits among patients with a visit, mean (SD)	11.9 (9.0)	3.7 (3.8)	<.001
Median	10.0	2.0	
Other outpatient visits*^a^*			
Patients with an other outpatient visit, n (%)	569 (99.8%)	1450 (84.8%)	<.001
Other outpatient visits among all patients, mean (SD)	35.2 (43.2)	5.5 (10.0)	<.001
Median	18.5	2.0	
Other outpatient visits among patients with a visit, mean (SD)	35.2 (43.2)	6.5 (10.5)	<.001
Median	19.0	3.0	
**Outpatient pharmacy**			
Patients with any prescription, n (%)	546 (95.8%)	1216 (71.1%)	<.001
Prescriptions among all patients, mean (SD)*^b^*	36.8 (32.1)	8.3 (14.8)	<.001
Median	29.0	2.0	
Prescriptions among patients with a prescription, mean (SD)*^b^*	38.4 (31.8)	11.7 (16.3)	<.001
Median	30.5	5.0	

Abbreviations: ER, emergency room; FH, familial hypophosphatemia; HCRU, healthcare resource utilization.

^*a*^Other outpatient visits included laboratory tests and imaging.

^*b*^Mean number of prescriptions per category was determined by the number of prescriptions for each patient and then averaged.

**Figure 2.**
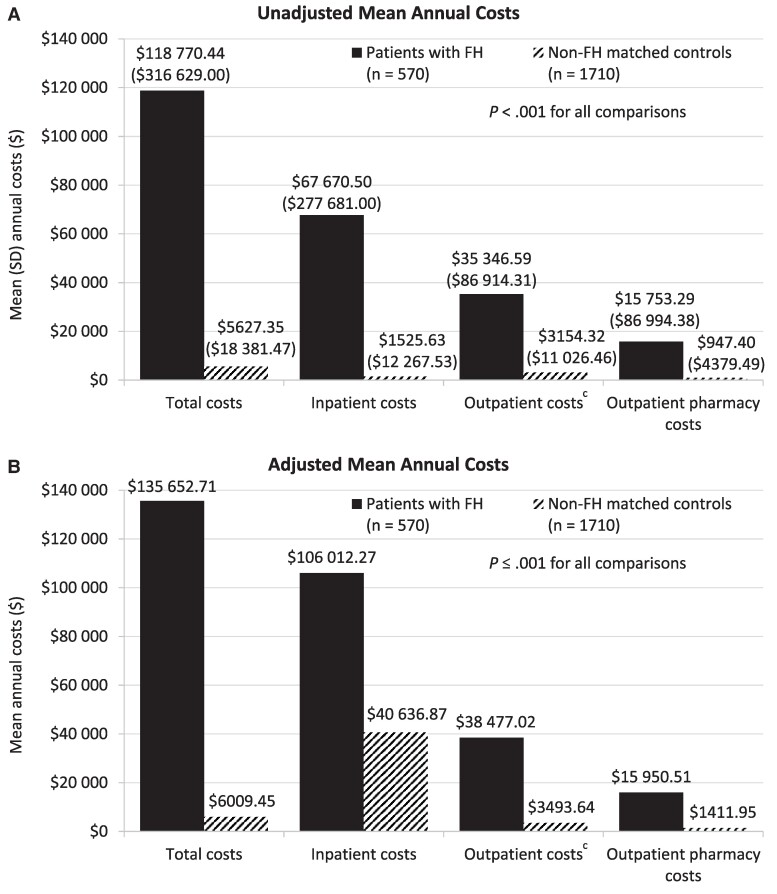
Unadjusted (A) and adjusted^a^ (B) mean all-cause healthcare costs during follow-up for patients with FH and non-FH matched controls^b^. Costs were calculated among all patients in each cohort. All costs were inflated to 2021 US dollars using the Medical Care Component of the consumer price index. ^a^Adjusted annual mean costs were estimated from two-part regression models, adjusting for age, sex, and insurance plan type. ^b^*P* < .001 for the comparison of unadjusted and adjusted annual mean all-cause costs between patients with FH and non-FH matched controls. ^c^Outpatient costs included costs associated with emergency room visits, outpatient office visits, and other outpatient services (eg, laboratory tests, imaging). Abbreviation: FH, familial hypophosphatemia.

Unadjusted mean (SD) all-cause healthcare costs were greater for patients with FH compared with controls (Fig. 2A), including 21.1-fold higher mean annual costs per patient for total healthcare services ($118 770.44 [$316 629.00] vs $5627.35 [$18 381.47]), 44.4-fold higher costs for inpatient admissions ($67 670.50 [$277 681.00] vs $1525.63 [$12 267.53]), 11.2-fold higher costs for outpatient services ($35 346.59 [$86 914.31] vs $3154.32 [$11 026.46]), and 16.6-fold higher costs for outpatient pharmacy prescriptions ($15 753.29 [$86 994.38] vs $947.40 [$4379.49]); all *P* < .001) (Supplementary Table S5) [37].

Mean all-cause healthcare costs remained higher among patients with FH than controls after adjustment (Fig. 2B; Supplementary Table S6) [37]. Patients with FH incurred 22.6-fold higher annual adjusted mean total healthcare costs per patient ($135 652.71 vs $6009.45, adjusted cost difference [ACD] = $129 643.26; *P* < .001). Differences in adjusted annual mean total costs for patients with FH compared with controls were driven by 2.6-fold higher all-cause inpatient admission costs ($106 012.27 vs $40 636.87; ACD = $65 375.40; *P* = .001), 11.0-fold higher outpatient service costs ($38 477.02 vs $3493.64; ACD = $34 983.38; *P* < .001), and 11.3-fold higher outpatient pharmacy prescription costs ($15 950.51 vs $1411.95; ACD = $14 538.56; *P* < .001).

### Dental Procedures, HCRU, and Costs

Overall, few dental procedures were reported among study participants identified in the MarketScan Dental database. Tooth extractions and root canals were performed in 5.6% and 2.8% of patients with FH, respectively (Supplementary Table S7) [37], and no participant had implants coded during the study period. Prophylactic routine dental cleaning was the most common type of procedure among both patients with FH and controls (36.6% vs 50.0%; *P* = .065), followed by fillings (18.3% vs 13.4%; *P* = .343). No significant differences in dental procedures were observed among patients with FH and controls when stratified by age (data not shown).

Utilization of any dental service was observed among > 60% of individuals in the MarketScan Dental database matched analysis (63.4% for patients with FH and 64.8% for controls) (Supplementary Table S8) [37]. Patients who obtained a dental service had approximately 7 services (mean of 6.8 dental services for patients with FH and 7.3 services for controls). Diagnostic services and oral and maxillofacial surgery were used by > 50% of patients with FH (57.7% for each) and controls (62.7% and 62.0%, respectively). The mean number of periodontal services and restorative services was lower among patients with FH than controls (1.1 vs 2.1; *P* = .020; and 1.4 vs 2.1; *P* = .045, respectively).

Total mean dental costs were numerically higher among controls compared with patients with FH, but did not differ significantly ($523.37 vs $402.76; *P* = .281) (Supplementary Table S9) [37]. Approximately half of dental costs among patients with FH were attributable to oral and maxillofacial surgery ($102.16) and restorative service ($104.77) costs, while costs among controls were primarily driven by restorative services ($129.05), oral and maxillofacial surgery ($100.89), and diagnostic service ($77.79) costs. No significant differences in dental services or costs were observed among patients with FH and controls when stratified by age (data not shown).

## Discussion

XLH, the most common form of FH, is associated with a substantial burden from pharmacological therapy use, ongoing disease and treatment monitoring, morbidities related to the disease and treatment, orthopedic procedures, and loss of productivity [2, 3, 5, 6, 12]. This real-world, retrospective cohort study quantified the patient and treatment characteristics, HCRU, and healthcare costs of burosumab-naïve patients with FH compared with matched controls without FH. The results provide valuable additional insights into the substantial clinical and healthcare resource burden of this rare disease.

This study focused on patients with FH as identified using the ICD-10 diagnosis code (E83.31) that covers this group of disorders. Although there is no specific ICD-10 diagnosis code to differentiate XLH from other forms of FH, most patients with the FH diagnosis code are expected to have XLH, given that this is the predominant form of the disorder. However, any diagnoses of FH coded in the study that were not XLH, including non-FGF23-mediated forms of hypophosphatemia, could not be excluded with the available data. Conversely, some patients with XLH may have been missed in our analysis if diagnoses of XLH were instead classified under other E83.3x diagnosis codes that broadly cover hypophosphatemia or other phosphorus metabolism disorders [39, 40].

After being matched on age, sex, geographic region, payer, and index year, the FH and control cohorts had similar demographic characteristics at baseline. Although FH often presents in childhood, most patients included in the study were adults, which might be attributable to the data available in the MarketScan administrative claims databases and to the study eligibility criteria, requiring ≥ 1 year of continuous enrollment pre-index, which excluded 687 out of 1977 patients with an FH diagnosis, and no use of burosumab, which excluded 78 out of 1977 patients, including 38 children. These criteria may have led to the exclusion of a greater proportion of pediatric patients with FH, who may not have had a lengthy pre-index enrollment period and who were more likely than adults to have received treatment with burosumab. The exclusion of patients treated with burosumab may also have led to a study population of less severely affected patients with FH. Additionally, because the MarketScan databases do not include Medicaid health coverage, more children with FH and more severe nonambulatory, nonworking adults in particular may have been excluded. Finally, given that patients with XLH spend a greater proportion of their lifespan as adults than as children, it is unsurprising that there would be more adults with the condition in the MarketScan databases.

Among the study population, patients with FH had substantially greater baseline comorbidity (CCI score) than controls, which was more pronounced with increasing patient age. The comorbidity burden in these patients supports previous research demonstrating that XLH-related morbidities and general comorbidities are more common in patients with XLH and related disorders than in controls [41, 42].

Following their index diagnosis, patients with FH in our study had higher proportions of morbidities compared with controls, and these morbidities and other complications generally increased with age. Renal disease was 11.1-fold more common in patients with FH than controls, delayed growth/difficulty in walking was 10.3-fold more common, and muscle weakness was 9.7-fold more common. In terms of general comorbidities, depression was 4.0-fold more common in patients with FH than controls. However, several reported morbidities, such as lower limb deformities, nephrocalcinosis, and fractures, occurred only in a small proportion of patients with FH in our study (3.5%, 0.7%, and 9.8%, respectively), whereas previous analyses suggest that a high proportion of patients with XLH have morbidities such as genu varum (63%-77%) and nephrocalcinosis (21%-70%) or experience fractures (9%-61%) [3, 5, 6, 12, 14, 31]. Unlike some previous studies that reported the history of events over a lifetime or over several years and involved survey data, our study was limited to a 12-month follow-up period as captured by claims data. Notably, ICD-10 diagnosis codes for some conditions such as genu varum and nephrocalcinosis may not have been frequently used by healthcare providers, reflecting potential coding bias and under-reporting in claims data. The low rates of coding for morbidities such as nephrocalcinosis might indicate a lack of detection, a lack of testing, or simply an absence of coding during follow-up after patients have had a condition confirmed by testing. Limited knowledge of rare diseases by healthcare professionals may also be a contributing factor, resulting in a lack of appropriate testing.

As expected, treatment use was also higher in our study in patients with FH than controls, with 20.4% of patients with FH (116 out of 570 patients) receiving treatment with vitamin D and/or phosphate salts during the follow-up period. Conventional treatment with active vitamin D and/or phosphate salts was prescribed in 14.6% of patients with FH, and nonactivated (native) forms of vitamin D were prescribed in 6.5% of these patients. While conventional treatment targets the active vitamin D and phosphate deficiencies resulting from excess FGF23 in patients with FH, native forms of vitamin D may also be prescribed to correct concomitant low vitamin D storage levels that can preclude the production of active vitamin D [1, 3, 6, 18, 41, 43]. Nevertheless, the use of conventional treatment or other forms of vitamin D in patients with FH in our study was very low, including in children. Previous multinational analyses of patients with XLH reported that conventional treatment with active vitamin D and/or phosphate salts was used in 41% to 100% of children and 30% to 84% of adults [5, 12, 33, 44]. The low treatment use observed in our study suggests that FH may not have been actively managed for many patients (especially adults) in this US-based claims database. As noted, the exclusion of burosumab may have also led to a study population of less severely affected patients with FH who required less treatment. Additionally, conventional treatment may not have been covered by the US insurance providers included in this study and thus would not have been detectable on claims data if patients were paying out of pocket.

All-cause opioid use was also greater for patients with FH compared with controls during the follow-up period (45.3% vs 11.7%). Some previous analyses have reported opioid use in approximately 20% of patients with XLH [14]. The higher use of opioids observed in our study may reflect the fact that the study population comprised mostly adults, or that even a single opioid prescription claim categorized patients as opioid users. Nonetheless, opioid use was also high in children with FH in our study (27.6% in patients aged 12-17 years; data not shown), possibly reflecting its use for postsurgical pain management. Additional studies are warranted to elucidate the reasons for chronic opioid use in patients with FH. In addition, antidepressant use was higher in patients with FH than controls in our study (40.7% vs 12.2%). This difference might reflect the higher prevalence of mental health issues in patients with FH, or the use of antidepressants to help manage chronic pain. Overall, the prevalence rates of depression, arthralgia, and myalgia in our study were significantly higher in patients with FH than controls (22.5% vs 5.6%, 25.4% vs 10.3%, and 4.6% vs 1.3%, respectively). A prior study in the United Kingdom likewise reported a higher prevalence of depression in patients with XLH than controls (25% vs 10%) [42].

Although previous studies have shown that appropriate treatment initiation early in childhood can reduce the severity of clinical symptoms of XLH experienced in adulthood, the symptoms of XLH that emerge in children often remain unresolved in adults [1, 3, 10, 12, 45-47]. The chronic effects of XLH thus signify the lifelong burden of this disorder, despite the use of conventional treatment in childhood [3, 4, 12, 14]. HCRU and associated clinical events have been reported to be higher in adult patients compared with pediatric patients, consistent with the progressive, lifelong nature of FH [5]. Consistent with previous evidence, all-cause HCRU in our study was high and was significantly greater for patients with FH than controls across all health service categories: 60.4% of patients with FH had all-cause inpatient admissions, 51.6% had outpatient emergency room visits, and 95.8% had outpatient pharmacy prescriptions. In terms of healthcare costs, mean all-cause total costs after adjusting for individual patient characteristics were approximately 23-fold higher for patients with FH compared with controls. Most of this difference was attributable to outpatient services and outpatient pharmacy prescription costs, each of which was approximately 11-fold higher in patients with FH compared with controls.

In the analyses of dental claims, there were surprisingly few significant differences between patients with FH and controls. Dental HCRU and costs remained similar among patients with FH and controls stratified by age group (data not shown). Although frequent dental complications are known to be associated with XLH, including dental necrosis, severe abscesses, periodontitis, and tooth loss [3, 8, 12], patients with FH may have avoidance issues with dental care or may not have access to professionals who have suitable knowledge of the disease and can provide appropriate care [48]. However, the dental outcomes in our study should be interpreted with caution, given the small sample size available from the MarketScan Dental database. Additionally, the dental burden for patients who utilized self-pay dental services would not be captured by the database. An estimated 50% of adults aged 18 to 64 years in the United States with private health insurance do not have dental coverage, and > 20% of those with dental coverage do not visit a dentist [49]. Among adults aged ≥ 65 years, approximately 70% do not have private or Medicare Advantage dental insurance [50]. Furthermore, some procedures, such as dental implants, may not be covered among those with insurance [51].

Overall, our study demonstrates that the clinical and healthcare resource burden for burosumab-naïve patients with FH is high compared with individuals without FH. The HCRU and cost burden in patients with FH likely reflects several factors, including the high occurrence of disease-related morbidities and the need for ongoing patient care from a multidisciplinary team [3, 5, 18, 42].

### Study Limitations

The longitudinal Merative MarketScan administrative claims databases used in this study provide information across the full continuum of care for a large sample of patients in the United States, enabling comprehensive evaluation of patient and treatment characteristics, HCRU, and associated costs. However, as a retrospective analysis involving administrative claims data, this study had several inherent limitations. Although the study population comprised individuals in the United States with a diverse payer mix of commercial health or private Medicare coverage as captured in the MarketScan claims databases, the results of this analysis may not be generalizable to patients with FH in other regions or with other insurance types or without health insurance coverage.

Although the cohorts were matched for age, sex, geographic region, payer, and index date, they could not be matched on race because this information was not available in the databases. Adjustments were further limited to those patient characteristics that could be measured from administrative claims in this real-world analysis. There may have been other, unidentified systematic differences between the study cohorts that accounted for observed differences. Additionally, as patients with FH were identified through administrative claims data rather than medical records, there was the potential for misclassification of FH status, covariates, or study outcomes; claims data are also subject to data coding limitations and data entry error. Given that FH is a group of genetic disorders requiring lifelong management, treatment approaches often focus on symptom alleviation and addressing morbid conditions, and the associated diagnosis codes may not appear as FH. As noted, the diagnosis of XLH and other forms of FH may have also been classified under other ICD-10 diagnosis codes that were not included in this study.

Conventional treatment for FH comprises active vitamin D and oral phosphate salts. As this study evaluated the use of both active and nonactivated (native) forms of vitamin D, the interpretation of conventional treatment use is limited. Given that this study involved claims data, we were also unable to assess nonprescribed sources of vitamin D. In addition, pharmacy prescription fills cannot determine whether a medication was taken as prescribed.

The morbidities analyzed in this study were frequently occurring conditions among patients with FH, but there may have been other potentially relevant conditions for this patient population that were not captured. Patients without dental coverage or for whom a procedure was otherwise self-pay (with or without coverage) were not captured in the MarketScan Dental database. Healthcare cost analyses also did not include all direct and indirect costs (eg, loss of productivity) associated with FH; further comprehensive evaluations of the economic burden of FH would be valuable.

Data were not available for other possible factors that may have influenced the observed outcomes, such as the presence of other prior treatment, FH disease severity or phenotype, and clinical response or occurrence of adverse effects during treatment. Finally, the exclusion of burosumab use may have limited the cohort to less severely affected children and adults, which would be expected to underestimate the burden of disease. Nevertheless, our study indicates a substantial clinical and healthcare resource burden with FH.

## Conclusions

Patients with FH had a greater clinical burden than matched controls, including baseline CCI score and medication use, and morbidities including renal disease over the 12-month follow-up period. All-cause HCRU and costs were substantially and significantly greater for patients with FH compared with controls. Future studies on the comparative HCRU and costs associated with conventional treatment and burosumab in pediatric and adult patients would be of interest as the treatment landscape for XLH and other FH disorders continues to evolve.

A plain language summary of this article is available in the supplementary material [37].

## Data Availability

Data supporting the findings of this study are available from the corresponding author upon reasonable request.

## Abbreviations

ACD: adjusted cost difference
CCI: Charlson Comorbidity Index
FGF23: fibroblast growth factor 23
FH: familial hypophosphatemia
HCRU: healthcare resource utilization
ICD: International Classification of Diseases
XLH: X-linked hypophosphatemia
